# High prevalence of HPV-56 and HPV-39 in Sari, Iran: an analysis of genotype distribution

**DOI:** 10.1186/s12985-024-02496-7

**Published:** 2024-09-20

**Authors:** Arash Letafati, Ali Vasheghani Farahani, Mohammad Mostafa Baradaran Nasiri, Hossein Pourmoein, Omid Salahi Ardekani, Haniyeh Ahoodashty, Mohammad Bagher Hashemi-Soteh, Aniseh Dadgar, Parisa Behshood, Iman Rezaee Azhar, Masoud Parsania

**Affiliations:** 1 Research Center for Clinical Virology, Tehran University of Medical Science, Tehran, Iran; 2 Department of Virology, Faculty of Public Health, Tehran University of Medical Sciences, Tehran, Iran; 3 Immunogenetic Research Center, Molecular and Cell Biology Research Center, Faculty of Medicine, Mzandaran University of Medical Sciences, Sari, Iran; 4 Department of Epidemiology and Biostatistics, School of Public Health, Tehran University of Medical Sciences, Tehran, Iran; 5 Department of Microbiology, Young Researchers and Elite Club, Shahrekord Branch, Islamic Azad University, Shahrekord, Iran; 6 Department of Microbiology, Faculty of Medicine, Tehran Medical Sciences, Islamic Azad University, Tehran, Iran

**Keywords:** Human papilloma virus, Genotype, Sexually-transmitted infections, Sari, Prevalence, Iran

## Abstract

**Background:**

Human papillomavirus (HPV) is responsible for the most common sexually transmitted infection, particularly among sexually active individuals. Understanding the geographical distribution and epidemiology of the most prevalent HPV genotypes is essential for developing effective prevention strategies. Consequently, this study aimed to examine the distribution of HPV genotypes among HPV-positive women and men in Sari, the capital city of Mazandaran province in northern Iran.

**Materials and methods:**

HPV DNA was extracted (PZP Company, Molecular IVD, Iran) from genital and cervical samples of the study participants. Genotyping was conducted for 90 cases utilizing the High + Low Papilloma Strip test (Operon Company, Spain). Demographic data were statistically analyzed in correlation with the virological data (STATA version 17).

**Results:**

Overall, 67.7% (61 out of 90) of the cases tested positive for HPV, with 75% of those being classified as high-risk. The participant group consisted of 92% females (83) and 8% males (7). The highest HPV prevalence, 75% (36), was observed in females and males aged under 31, with the majority of positive cases belonging to high-risk genotypes. The most frequently identified genotypes were HPV-11 (23%), HPV-6 (21%), HPV-56 (18%), HPV-39 (16%), HPV-16, HPV-91, and HPV-66 each comprising (14%). HPV-56 was the most common high-risk genotype, accounting for 11 cases (18%), followed by HPV-39, which was present in 10 cases (16%).

**Conclusion:**

The prevalence of HPV infection was particularly high among individuals under the age of 31 for both genders, with men exhibiting a 100% infection rate. These findings emphasize the urgent need for targeted education aimed at the younger population and the implementation of infection control measures. Specifically, widespread HPV vaccination targeting HPV-6, HPV-11, HPV-39, and HPV-56 should be prioritized for the general population.

## Introduction

Human Papillomavirus (HPV), a member of the *Papillomaviridae*, possesses circular double-stranded DNA and is divided into two groups: high-risk (HR) HPVs and low-risk (LR) HPVs [1, 2]. HR-HPVs are associated with several types of cancer, including anogenital and oropharyngeal cancers, such as anal, vulvar, vaginal, cervical, and penile cancers [3, 4]. Conversely, LR-HPVs primarily manifest as cutaneous and anogenital warts [5]. Genital infections caused by HPV are the prevailing sexually transmitted infections worldwide, affecting up to 90% of sexually active women. The prevalence varies based on factors such as geographical region, population source, and detection methodology [6, 7]. Currently, more than 150 genotypes of HPV have been identified, with approximately 40 known to be sexually transmitted and capable of infecting the anogenital region [8]. The International Agency for Research on Cancer (IARC) sorts HPV into categories by their cancer-causing potential: group 1 (carcinogenic), group 2 A (probably carcinogenic), group 2B (possibly carcinogenic), group 3 (low risk), and unclassified. Types such as HPV-16, -18, -31, -33, -35, -39, -45, -51, -52, -56, -58, and − 59 are also considered carcinogenic to humans. HPV-68 is deemed probably carcinogenic, while HPV-26, -30, -34, -53, -66, -67, -69, -70, -73, -82, -85, and − 97 are possibly carcinogenic. Low-risk types include HPV-6, -11, -42, -43, and − 44 [9].

High-risk strains of HPV are more likely to lead to cancer compared to others, although the progression of infection to this stage can take time. On the other hand, certain HPV strains are less harmful, targeting the skin and mucous membranes and leading to issues like warts, unusual growths, or non-cancerous tumors. The impact of an HPV infection is influenced by numerous factors, including environmental conditions, the characteristics of the host, and the nature of the virus. People with compromised immune systems are more susceptible to infection than those with robust health [10]. Many HPV infections are frequently asymptomatic and tend to resolve on their own within two years, generally without needing medical treatment. Nonetheless, ongoing infections with high-risk HPV types can result in precancerous changes and eventually cancer. Additionally, studies show that HPV is responsible for most cases of cervical cancer and various other malignancies in the genitourinary tract and throat [11, 12].

Extensive research has indicated a strong correlation between HPV infection and cervical cancer as well as precancerous lesions in women of Asian descent. These findings emphasize the significance of HPV as a factor to the development of cervical cancer in this particular population [13]. Approximately half of cervical cancer cases are caused by high-risk strains of HPV, particularly HR-HPV16, while HR-HPV18 accounts for about 20% of these cases [14]. Cervical cancer ranks as the fourth most prevalent cancer among women worldwide, with a particularly high incidence in low- and middle-income countries (LMICs) like Brazil, India, China, South Africa (SA), and Iran. These countries face a substantial burden of cervical cancer cases [15]. Additionally, statistics show that in 2020, there were about 604,000 new cases and 342,000 deaths from cervical cancer worldwide [16, 17].

Currently, there are six approved prophylaxis options available, each offering distinct protection against HPV. Among these options, there are three bivalent vaccines, two quadrivalent vaccines, and one non-valent vaccine [18]. The initial trials conducted on the renowned GARDASIL and Cervarix vaccines demonstrated their remarkable effectiveness in safeguarding against incidental HPV infections and grade 3 cervical intraepithelial neoplasia. GARDASIL, a quadrivalent vaccine, contains HPV-16, HPV-18, HPV-6, and HPV-11 VLPs (virus-like particles), while Cervarix, a bivalent vaccine, contains HPV-16 and HPV-18 VLPs. Furthermore, there is a nonavalent vaccine known as Gardasil-9, which protects a wider range of HPV genotypes, including 6, 11, 16, 18, 31, 33, 45, 52, and 58. These vaccines serve as crucial tools in preventing HPV-related diseases and promoting overall health and well-being. Notably, GARDASIL has also shown efficacy in protecting against vaginal/vulva lesions and genital warts. Although there is limited data on newer HPV vaccines, a promising randomized phase III trial conducted on two Chinese vaccines, a quadrivalent vaccine (4vHPV) and a nine-valent vaccine (9vHPV), demonstrated comparable safety and efficacy to GARDASIL. Moreover, GARDASIL9 now includes indications for cancers of the oropharynx and other head and neck cancers caused by specific HPV types, demonstrating a significant improvement in the vaccine’s effectiveness [18].

In Iran, cervical cancer is not ranked among the top 10 cancers affecting females, yet it is estimated to have an annual incidence of approximately 947 cases. Despite its relatively low incidence compared to other cancers, cervical cancer remains a significant public health concern in Iran, emphasizing the importance of improving screening and prevention efforts [19]. Given the importance of HPV prevalence and the presence of high-risk strains, our study aimed to evaluate the incidence of HPV infections and identify the associated genotypes, including both high-risk and low-risk variants, in the capital city of Mazandaran province in northern Iran. This study focused on a cohort of individuals to investigate the prevalence of HPV infection among patients who tested positive for HPV.

## Materials and methods

### Study population

This research, carried out from August 2019 to March 2021, was a collaborative effort with the Clinical Virology Research Center (RCCV) at Tehran University of Medical Sciences. It included a total of 90 genital and cervical samples from male and female participants, collected from laboratories in Sari city. Samples were collected from female patients by brushing the cervix, while for males, samples were obtained by scratching, typically involving scraping or swabbing the area around the penis, including the urethra and genital skin. All participants gave informed consent and did not receive any medical treatments. In routine cytology evaluations, doctors sent samples, including Pap-Preps (liquid-based cytology) and cervical secretions, to laboratories in line with established cervical cancer screening guidelines. For individuals not following regular screening practices, such as virgins, samples were taken from cervical and vaginal secretions by physicians or skilled lab personnel to identify HPV. These samples were kept at 4 °C until they were tested the following day.

The research criteria involved women who, after a Pap smear, opted for further examinations following specialist recommendations. Additionally, it encompassed women advised by their healthcare provider to undergo testing due to the detection of conditions such as cervical cancer, breast cancer, and tumors in the head and neck region. For men, the criteria consisted of engaging in risky sexual behavior or having a partner infected with HPV, which motivated them to undergo HPV testing to confirm their health status. All demographic data were obtained from laboratory records and then transferred to the RCCV for additional analysis.

### DNA extraction and PCR

Preparation of samples before amplification, along with DNA extraction and HPV genotyping, were conducted within the molecular genetics department of the laboratory. Procedures adhered to protocols established by the quality control supervisor and were overseen by RCCV. For HPV-DNA extraction we utilized the PZP Company Molecular IVD, Iran total DNA extraction kit, following the manufacturer’s guidelines. Genotyping was done using the High + Low Papilloma Strip test by Operon, a company based in Zaragoza, Spain. This test can identify 19 high-risk HPV genotypes, including 16, 18, 26, 31, 33, 35, 39, 45, 51, 52, 53, 56, 58, 59, 66, 68, 69, 73, and 82, as well as 18 low-risk HPV genotypes, including 6, 11, 40, 42, 43, 44, 54, 61, 62, 67, 70, 71, 72, 74, 81, 83, 84 and 91.

### Statistical analysis

In this study, we used STATA (version 17) for data analysis. We calculated the ratio of positive response to the infection test in all people and in separate age and gender groups, then we compared the results of two gender groups with an independent t test, and to compare the relationship between age group and infection rate, we calculated the odds ratio (OR). In all tests, we consider the first type error level to be 0.05.

For those who refer to the mentioned medical centers, 30 different HPV genotypes have been tested, and anyone who responds positively to the test of one or more types of HPV is considered infected. In this sample of 90 people 26 genotypes were observed. People are divided into 4 age groups and patients are found in all ages. After presenting the statistical analysis of the collected data, we verified the reliability and accuracy of the results using statistical methods.

## Results

### Demographic data

The frequency and percentage of positive and negative HPV in each subgroup of gender and age can be seen in Table 1. Participants were categorized into 4 groups based on age. Out of 90 patients, 83 (92%) were women and 7 (8%) were men. In the subgroup of women, out of 83 cases, 54 (65%) were HPV positive and 29 (35%) were HPV negative. All referred men (7 people) were HPV positive (Table 1).

We conducted a t-test to compare the percentage of patients in the group of women and men, and the results are as follows: the difference between the two groups percentages is 35%. To test the difference between the ratio of women and men against equality, P-Value = 0.03 and the assumption of more infection in men referred for testing is confirmed.

**Table 1:** Distribution of observations categorized by gender and age across all participants

		HPV Positive	HPV Negative	Total
gender	Female	54 (65%)	29 (35%)	83 (92%)
	Male	7 (100%)	0 (0%)	7 (8%)
	< 31	36 (75%)	12 (25%)	48 (53%)
Age	31–40	16 (57%)	12 (43%)	28 (31%)
	41–50	7 (70%)	3 (30%)	10 (11%)
	50<	2 (50%)	2 (50%)	4 (5%)
	Total	61 (68%)	29 (32%)	90

Additionally, participants are divided into four age groups. The first group consists of individuals aged 30 and below, which includes one person aged 19, with the remaining participants being between 20 and 30 years old. This group includes 48 people, of whom 36 (75%) had a positive test and 12 (25%) were negative. Likewise, the frequency and percentage of other age groups are shown in Table 1. The largest group of people who refer for the test are the first age group, and this category also has the highest percentage of positive test results.

To compare the rate of infection in age groups, we used the odds ratio (OR) test, and we considered the group of 50 years and above, which had the lowest rate of infection, as the reference group. The results are listed in Table 2. For the age group less than 31 years old, the number obtained is 3, which means that the chance of being positive for HPV in the age group under 31 years old is 3 times that of the group over 50 years old, and this ratio is significant (P-value = 0.001).

**Table 2:** Odds ratio of HPV positive between age groups

		OR	*P*-Value
	< 31	3.0	0.001
Age	31–40	1.3	0.451
	41–50	2.3	0.220
	50<	reference	

In the same way, the OR in the other two age groups is 1.3 and 2.3, but according to the P-values, we see that at the error level of 0.05, these ratios are not significant in the other two age groups.

We calculated the OR in different age groups with a reference group (group under 20 years). For example, for the 21–30 age group, the number obtained is 2.103 (P-value = 0.509), which means that the chance of being positive for HPV in the 21–30 age group is 2.1 times compared to the age group under 20 years. But according to the P-values, we see that these ratios are not significant in any group at the 0.05 error level. We do not calculate and test this odds ratio for the gender group, because due to the extreme difference in the sample size between men and women, this test will not be accurate.

### Distribution of HPV genotypes

**Fig. 1:**
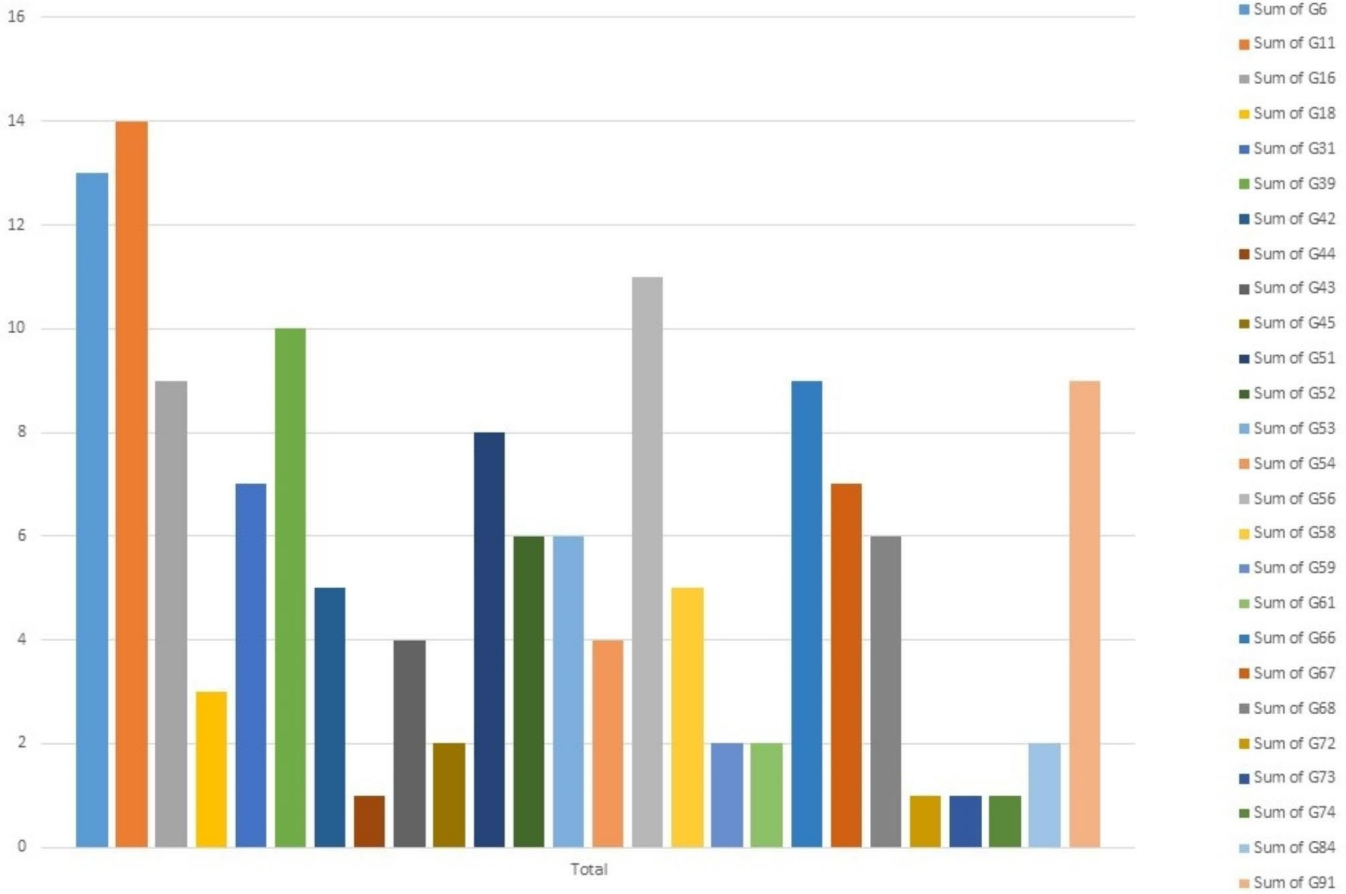
Frequency chart of all HPV genotypes. The distribution of HPV genotypes is as follows: HPV-11 with 14 cases (23%), HPV-6 with 13 cases (21%), HPV-56 with 11 cases (18%), HPV-39 with 10 cases (16%), HPV-91, 66, and 16 each with 9 cases (14%), HPV-51 with 8 cases (13%), HPV-31 and 67 each with 7 cases (11%), HPV-52, 53, and 68 each with 6 cases (9.8%), HPV-58 and 42 each with 5 cases (8%), HPV-54 and 43 each with 4 cases (6.5%), HPV-18 with 3 cases (5%), HPV-45, 59, 61, and 84 each with 2 cases (3.2%), and HPV-74, 73, 72, and 44 each with only 1 case

**Fig. 2:**
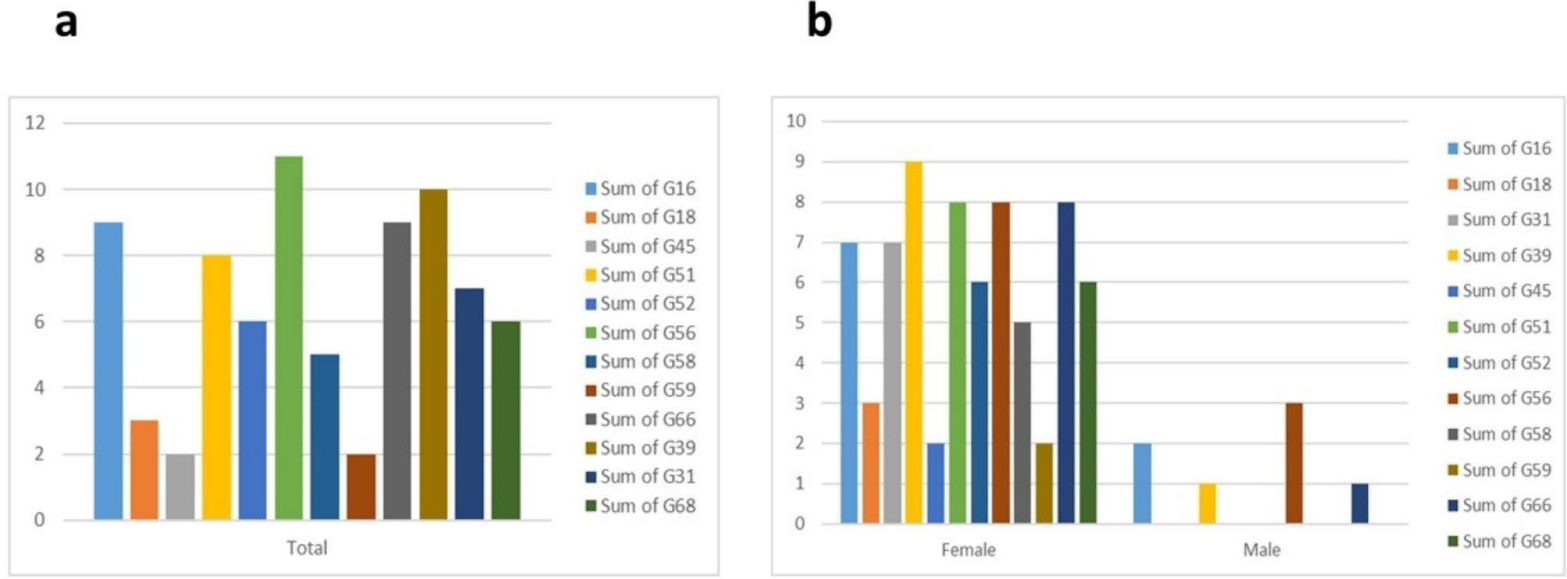
Diagram of the High-Risk genotypes including type 16, 18, 31, 39, 45, 51, 52, 56, 58, 59, 66 and 68 (**a**) In the whole sample, (**b**) By gender

26 different genotypes were identified among the people referred for HPV diagnosis, and anyone who tested positive for one of these genotypes was diagnosed as infected. The distribution of each genotype’s is depicted in Fig. 1. The most abundant genotypes are, 11 (23%), 6 (21%) and 56 (18%), 39(16%) respectively. HPV-74, 73, 72, and 44 each have just one case, representing the lowest occurrence frequency. The frequency chart of this type, as well as the frequency by gender, is shown in Fig. 2.

Genotypes 16, 18, 31, 39, 45, 51, 52, 56, 58, 59, 66, and 68 are classified as high-risk types. Their frequency distribution is detailed in Table 3 and illustrated in Fig. 2. HPV-positive cases are categorized as HR if they are positive for any of the listed genotypes, and as LR if they are not positive for any of these genotypes. The most common HR genotypes are type 56, with 11 cases (18%), and type 39, with 10 individuals (16%). Conversely, the least frequent HR genotype is type 59, with only 2 individuals (3%). Moreover, the LR genotype with the highest frequency includes type 11, with 14 occurrences (23%), and type 6, with 13 individuals (21%). In the context of co-infection with multiple genotypes, 39 subjects had co-infection among infected individuals which is near 61.3%. Highest detected genotype among this mixed infection were HPV-6 (13/61 cases), HPV-11 (14/61 cases), HPV 56 (11/61 cases) and HPV 39 (10/61 cases) respectively.

**Table 3:** HR HPV types identified in the study and assigned by IARC as having an oncogenic risk

HR Genotype*	Frequency	percent
HPV- 16	9	15%
HPV- 18	3	5%
HPV- 31	7	11%
HPV- 39	10	16%
HPV- 45	2	3%
HPV- 51	8	13%
HPV- 52	6	10%
HPV- 56	11	18%
HPV- 58	5	8%
HPV- 59	2	3%
HPV- 66	9	15%
HPV- 68	6	10%

* This table indicate that these 12 HPV types are designated by the international agency for research on cancer (IARC) as group 1 carcinogens

Table 4 provides details on HR and LR HPV genotypes. In the HR column, it shows the frequency of the type of high-risk disease in gender and age subgroups. For example, among infected women, 40 people (74%) and 10 people (62%) in the age group of 31 to 40 years have the high-risk type.

**Table 4:** Frequency of HR and LR genotypes by age and gender and their percentage among HPV positive people

		HR	LR	HPV Positive
gender	Female	40 (74%)	14 (26%)	54
	Male	6 (86%)	1 (14%)	7
	< 31	29 (81%)	7 (19%)	36
Age	31–40	10 (62%)	6 (38%)	16
	41–50	6 (86%)	1 (14%)	7
	50<	1 (50%)	1(50%)	2
	Total	46 (75%)	15 (25%)	61

## Discussion

Many HPV genotypes are known to play a significant role in the development of cervical cancer (CC), with nearly all cases of cervical cancer being associated with these types [20]. Additionally, this virus has also been recognized as a causative agent for other cancers such as anal, genital, vaginal, oropharyngeal, and head and neck cancer (HNCs) [21, 22]. The prevalence of this virus among sexually active Iranians is growing, regardless of economic, marital, or age-related factors [23–25]. Our investigation reveals the low presence of a High-risk HPV-18 genotype among positive cases, emphasizing a distinctive pattern of high-risk HPV genotypes within this city in comparison to other districts in Iran. On the contrary, HPV-39 and HPV-56, were reported more frequently among this population. Therefore, the investigation of epidemiological patterns regarding the genotypes of the virus has the aptitude to be very beneficial in creating effective programs for the prevention and appropriate accomplishment of HPV-related cancers, especially cervical cancer.

This study’s findings indicate that 68% of the participants were found to have at least one genotype of HPV, which is higher compared to the reported prevalence rates of 7.8% (of the 825 female), 20.1% (of the 308 female), and 45.4% (of the 436 female) in previous studies [26–28]. Furthermore, the occurrence of high-risk genotypes (75%) was nearly three times higher than that of low-risk genotypes (25%) in our study. Conversely, a study conducted in northeastern Iran between 2016 and 2018 conveyed a lower ratio of high-risk genotypes (32.03%) to low-risk genotypes (17.41%) which was less than two times [29]. Similar to the current study, another study in Iran, showed a ratio of almost three times lower for high-risk HPV genotypes (74%) compared to low-risk HPV genotypes (26%) [30]. Considering that the prevalence of HPV genotypes in individuals under 31 years old was reported to be higher (75%), it is noteworthy that a study involving 10,266 Iranian males and females found the highest incidence of HPV in the age group 30–44 years old (51.8%) [31]. A study conducted between 2011 and 2012 examined 1,218 Iranian women aged 16–60 to determine the prevalence and types of HPV, as well as the factors influencing HPV infection risk in women with cervical cytology. The study found higher prevalence rates in women under 20 (11.3%) and over 50 (8.6%) [32]. In contrast, our observation showed the highest HPV prevalence in individuals under 31, with most positive cases being high-risk genotypes.

Another research highlights HPV-11 (23%) and HPV-6 (21%) as the most frequently identified low-risk HPV genotypes. Also, a study involved 134 patient samples (including 127 patients referred to gynecological clinics and 7 patients with solid cervical tumors), to determine the most common HPV genotypes in women whose cervical cytology was normal or abnormal, HPV-6 and HPV-11 genotypes were reported (8%) as common genotypes [33]. Likewise, in the study conducted in South Khorasan, Iran between 2014 and 2015, 253 random pap smear samples from women were examined to determine the frequency and genotypes of HPV in women with normal and abnormal cytology. The most common genotypes were HPV-6 (64%) and HPV-11 (24%) [34]. On the other hand, a study aimed at providing information on the genotypes and prevalence of HPV infection in Kermanshah, Iran, analyzed 87 samples, including cervical samples from women with genital warts and skin biopsy samples from men. The prevalence of the HPV-6 genotype was found to be low and detected only in cases of co-infection with HPV-50s, HPV-30s, HPV-16, and HPV-18 genotypes [35]. In contrast, our findings indicated that the most common genotypes were HPV-11 (23%) and HPV-6 (21%).

Contrary to this study’s findings that HPV-56 (18%) and HPV-39 (16%) were among the most common high-risk genotypes, a study conducted in Iran from 2019 to 2021 involving 5,176 cases revealed a low prevalence of these genotypes. Out of 2,727 (53%) HPV-positive cases, HPV-56 and HPV-39 accounted for only 3% and 2%, respectively [23]. HPV-18 exhibits a low prevalence (5%) and is not among the most common high-risk genotypes, in contrast to previous studies where it was reported as one of the most common genotypes [36–39]. In line with our results, during the study period from 2022 to 2023 in Sanandaj, Iran, which evaluated 950 people for the occurrence and spread of HPV genotypes, HPV-18 was not among the most common genotypes and other high-risk genotype such as HPV-16 (17%) and HPV-52 (13%) were most common [40]. Furthermore, in the investigation examining HPV prevalence in the oral cavity in southeastern Poland, HPV-18 was detected in adults in only 0.8% of cases (2 samples), representing the lowest frequency [10]. Although HPV-16 was not the most prevalent genotype in our study, previous research shows that it continues to have significant prevalence [41–44]. HPV-45 and HPV-59 genotypes reported a low prevalence (both 3%) similar to our study results.

In our study, we observed that HPV types 11 and 6 were most prevalent among the LR group, while HPV types 56 and 39 were most common in the HR group. In Iran, HPV vaccines are provided by private entities, and insurance coverage is limited or nonexistent. This situation makes the vaccine costly and less accessible for many individuals [45, 46]. Furthermore, HPV vaccination is not included in the national immunization schedule, which further limits its reach [47]. Currently, the available vaccines in Iran are the bivalent and quadrivalent vaccines. The bivalent vaccine covers HPV types 16 and 18, which the quadrivalent vaccine targets HPV types 6, 11, 16 and 18 [48, 49]. Our study found that while these vaccines protect against some of the prevalent HPV types, they do not cover all HR genotypes. Particularly, HPV types 56 and 39, which are prevalent in our study population, are not included in the current vaccine offerings.

Given the limitations of the current vaccines and the prevalence of these HR HPV types, there is a clear need for measures to improve vaccine coverage. Expanding the range of HPV types included in the vaccines could provide more comprehensive protection. Additionally, preventive measures and educational programs could play a crucial role in increasing awareness about HPV and the importance of vaccination. Such initiatives could help address gaps in vaccine coverage and improve public health outcomes. In conclusion, based on our study results and the current vaccine availability in Iran, the existing vaccines primarily protect against low-risk and some high-risk HPV genotypes. However, other prevalent high-risk genotypes, such as HPV types 56 and 39, remain uncovered. There is a pressing need for enhanced vaccine coverage and public education to better safeguard against HPV-related diseases and to address the current limitations in vaccine accessibility.

Additionally, it is important to recognize several limitations encountered in this study. The reported prevalence of HPV in patients may not accurately depict the true HPV epidemiology within the community. Additionally, larger studies with expanded populations, particularly focusing on men, are essential for gaining a comprehensive understanding of HPV infection prevalence and its impact across diverse demographics. Given the clinical significance of HPV, future research should increase sample sizes and encompass various geographical regions within the country.

## Conclusion

Our research found a 67.7% prevalence of HPV infection and analyzed the types of HPV present, revealing that 75% of high-risk HPV genotypes were detected in both Iranian men and women living in Sari. The high prevalence of HR-HPV subtypes, especially HPV-56 and HPV-39, highlights the urgent need for proactive measures.

Accurate analysis of circulating HPV types is essential for advancing cancer prevention and healthcare. By identifying and monitoring the prevalent HPV types in our study population, we contribute to understanding their role in cancer development and informing targeted diagnostic and preventive strategies. The detection of these high-risk genotypes emphasizes the need for enhanced HPV vaccination and awareness initiatives. We recommend launching an HPV vaccination and awareness program specifically targeting individuals under 31 years old, who exhibited a notable prevalence of HPV infection in our research. This focused approach has the potential to substantially reduce the incidence of HPV-associated cancers and improve public health outcomes in the region. In conclusion, our study highlights the importance of continuous analysis of HPV types to tailor effective prevention strategies. Addressing the gaps in vaccine coverage and implementing targeted preventive measures will help manage HPV-related health issues more effectively and reduce the risk of HPV-related cancers.

## Data Availability

Data will be made available on reasonable request from corresponding author.

## Declarations

### Use of an LLM

We utilize Large Language Models (LLMs), such as ChatGPT, exclusively for enhancing the quality of grammar while maintaining ethical standards.
